# Impact of the Graphene Production Methods Sonication and Microfluidization on *In Vitro* and *In Vivo* Toxicity, Macrophage Response, and Complement Activation

**DOI:** 10.1021/acsomega.4c03189

**Published:** 2024-09-18

**Authors:** Jan-Lukas Førde, Abdelnour Alhourani, Tian Carey, Adrees Arbab, Kari E. Fladmark, Silje Skrede, Tom Eirik Mollnes, Lars Herfindal, Hanne R. Hagland

**Affiliations:** † Department of Internal Medicine, Haukeland University Hospital, 5009 Bergen, Norway; ‡ Centre for Pharmacy, Department of Clinical Science, University of Bergen, 5020 Bergen, Norway; § Department of Chemistry, Bioscience and Environmental Engineering, University of Stavanger, 4021 Stavanger, Norway; ∥ Textile Two Dimensional Ltd., London WC2H 9JQ, England; ⊥ Department of Biological Sciences, University of Bergen, 5020 Bergen, Norway; # Section of Clinical Pharmacology, Department of Medical Biochemistry and Pharmacology, Haukeland University Hospital, 5021 Bergen, Norway; ∇ Department of Clinical Science, University of Bergen, 5020 Bergen, Norway; ○ Research Laboratory, Nordland Hospital Trust, 8092 Bodø, Norway; ◆ Department of Immunology, Oslo University Hospital and University of Oslo, 0372 Oslo, Norway; †† School of Physics, CRANN & AMBER Research Centre, Trinity College, Dublin, 2, Ireland

## Abstract

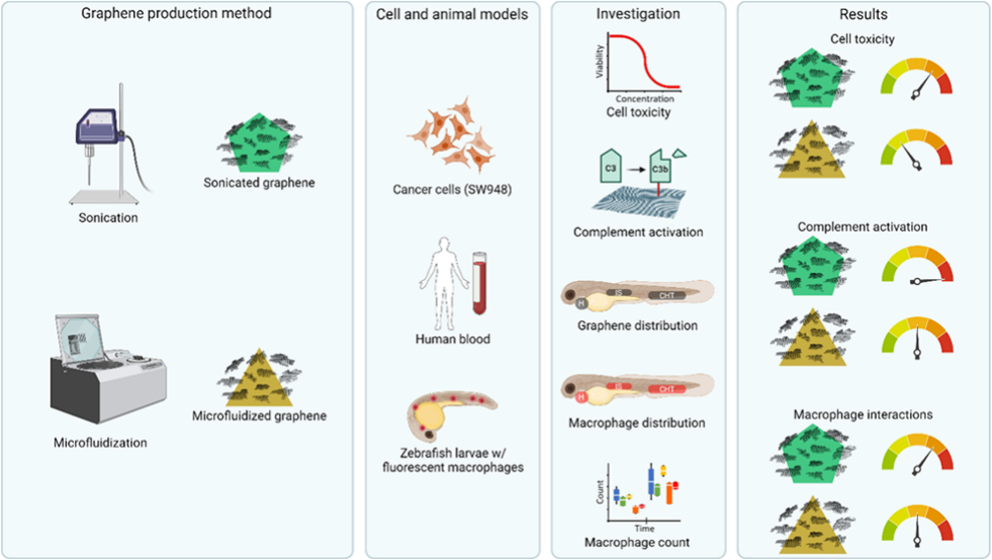

Graphene, a material composed of a two-dimensional lattice of carbon atoms, has due to its many unique properties a wide array of potential applications in the biomedical field. One of the most common production methods is exfoliation through sonication, which is simple but has low yields. Another approach, using microfluidization, has shown promise through its scalability for commercial production. Regardless of their production method, materials made for biomedical applications need to be tested for biocompatibility. Here, we investigated the differences in toxicity, macrophage response, and complement activation of similar-sized graphene flakes produced through sonication and microfluidization, using *in vitro* cell assays and *in vivo* assays on zebrafish larvae. *In vitro* toxicity testing showed that sonicated graphene had a high toxicity, with an EC_50_ of 100 μg mL^–1^ for endothelial cells and 60 μg mL^–1^ for carcinoma cells. In contrast, microfluidized graphene did not reach EC_50_ at any of the tested concentrations. The potency to activate the complement system in whole blood was 10-fold higher for sonicated than for microfluidized graphene. In zebrafish larvae, graphene of either production method was found to mainly agglomerate in the caudal hematopoietic tissue; however, no acute toxic effects were found. Sonicated graphene led to an increase in macrophage count and a macrophage migration to the ventral tail area, while microfluidized graphene led to a transient reduction in macrophage count and fewer cells in the ventral trail area. The observed reduction in macrophages and change in macrophage distribution following exposure to microfluidized graphene was less pronounced compared to sonicated graphene and contributed to masking of the fluorescent signal rather than cytotoxic effects. Summarized, we observed higher toxicity, macrophage response, and complement activation with graphene produced through sonication, which could be due to oxygen-containing functional groups introduced to the edge of the carbon lattice by this production method. These findings indicate that microfluidization produces graphene more suitable for biomedical applications.

## Introduction

Graphene is a nanomaterial comprised of a single sheet of carbon atoms arranged in a hexagon lattice and has, together with its derivatives such as few-layered graphene and graphene oxide, gained interest for many biomedical applications such as biosensors, cell scaffolds, and as a drug carrier through its capability to bond aromatic drugs with a pH-dependent release.^1,2^ When deployed in the human body, nanoparticles have two main routes of clearance. Particles between 10 and 20 nm are cleared through renal filtration, while larger particles can trigger activation of the complement system, a series of proteins that make up part of the immune system, and get marked for phagocytosis by the monocyte–macrophage system.^3^ Activation of the complement system not only facilitates phagocytosis through opsonization but can also lead to undesired inflammatory responses.^4^ Hence, biomedical materials must be carefully chosen to prevent, or at least minimize, the activation of the complement system.

For laboratories, a convenient and frequently used approach for graphene production is the exfoliation of layers from graphite through sonication.^5,6^ While this production method is suitable for research laboratories, low yields and long production time make it less applicable for large-scale production. Additionally, the process of sonication has been linked to the formation of defect sites containing oxygen-rich functional groups, which, in turn, could lead to increased cytotoxicity and complement system activation.^7−9^ Another graphene production method is exfoliation using microfluidization.^6^ In this process, a graphite suspension is forced through microchannels by a high-pressure pump. When passing through the interaction chamber, high shear forces exfoliate a single or few layers of graphene from graphite. This process can be repeated to exfoliate any unexfoliated remaining graphite. Using this method, graphene concentrations of up to 100 g L^–1^ and a 100% mass exfoliation yield and throughput of ∼9.3 g h^–1^ have been demonstrated.^6^

To investigate whether graphene produced through microfluidization has improved potential for biomedical applications, such as drug delivery, a suitable test model is needed. While *in vitro* experiments offer valuable insight into a single cell basis, they fail to accurately represent the environment and interactions found in tissues, organs, and the whole organism. *In vivo* models, such as rodents, may serve as models for toxicological studies on an organismal level, but on the level of tissues and individual cells, toxicological mechanisms in live animals can be challenging to monitor. Using the larvae of zebrafish (*Danio rerio*) as a toxicological model offers a good compromise between *in vitro* cell studies and *in vivo* studies in rodents.^10^ Zebrafish only require 2 days from fertilization to the development of a functioning circulatory system and completion of initial organogenesis, making them ideal for *in vivo* toxicity screening.^11^ Due to external fertilization, genetic manipulation is easily achieved through the injection of genetic material at the one-cell stage.^12^ A zebrafish strain highly suitable for the observation of fluorescently labeled cells is casper.^13^ The casper strain is a cross of two mutants, resulting in the lack of both iridophores and melanocytes, thus facilitating the obstruction-free imaging of the entire organism by using optical microscopy.

The current research aimed to compare the toxicity and immunoreactivity of graphene produced through sonication and microfluidization, using cell lines to evaluate cytotoxicity, human blood to investigate complement activation, and zebrafish larvae with fluorescent macrophages as an *in vivo* model.

## Materials

Graphite flakes for sonication, carboxymethylcellulose sodium salt (CMC), sodium deoxycholate (SDC), penicillin, streptomycin, and methanesulfonate (Tricaine) were obtained from Merck Life Sciences (Darmstadt, Germany). For microfluidization, graphite flakes were obtained from Imerys (Paris, France). The cell medium Dulbecco’s modified Eagle’s medium (DMEM) with 2 mM l-glutamine was purchased from Corning (Corning, NY). Both endothelial cell growth medium-2 (EGM-2) and the EGM-2 Endothelial SingleQuots Kit were purchased from Lonza (Basel, Switzerland). Fetal bovine serum (FBS) was purchased from BioWest (Nuaillé, France). Microinjection pipets for graphene injection (VESbv-11-0-0-55) were from BioMedical Instruments (Zöllnitz, Germany). The cell lines SW948 epithelial colorectal cancer cells were sourced from the European Collection of Authenticated Cell Cultures (Salisbury, U.K.) and human umbilical vein endothelial cells (HUVECs) acquired from Lonza (Basel, Switzerland)

## Methods

### Graphene Production

Three different graphene samples were produced for this work. The name for each sample is prefixed by G for graphene, followed by the production method of sonication (S) or microfluidization (M), and ending with the stabilization agent, carboxymethylcellulose sodium salt (CMC) or sodium deoxycholate (SDC).

G-S-CMC was made by dispersing 10 mg mL^–1^ of graphite flakes and 3 mg mL^–1^ of CMC in deionized water at pH 6 and left overnight in a fume hood to dissolve. The dispersion was then sonicated for 9 h using a Fisherbrand FB15069 (Fisher Scientific, Waltham, MA) to enable the exfoliation of graphite to graphene. Using a Sorvall WX100 mounting a TH-641 swinging bucket rotor (Thermo Scientific, Waltham, MA), the suspension was centrifuged at 20,000 rcf for 20 min to discard the sediment and remove unexfoliated graphite. The supernatant was used as the G-S-CMC graphene sample.

G-M-CMC was made by dispersing 100 mg mL^–1^ of graphite flakes with deionized water at pH 6 and 3 mg mL^–1^ of CMC and left overnight in a fume hood to dissolve the CMC. For the production of microfluidized graphene, the graphite concentration was increased by a factor of 10 due to the comparatively higher sheer rate. A schematic of this system can be found in a publication by Karagiannidis et al.^6^ If the same graphite concentration was used for sonicated graphene, it is likely that the majority would remain unexfoliated and would be lost during the centrifugation step. The graphite and CMC mixture was processed with an M-110P microfluidizer (Microfluidics International Corporation, Westwood, MA) with a Z-type geometry interaction chamber with microchannels of ∼87 μm wide for 100 cycles at 207 MPa system pressure. One cycle is defined as a complete pass through the rotor chamber.

G-M-SDC was made by dispersing 100 mg mL^–1^ of graphite flakes with deionized water at pH 6 and 5 mg mL^–1^ of SDC. The mixture was processed under the same conditions as G-CMC-M in a microfluidizer for 100 cycles.

Following graphene production, the graphene samples were ensured to be free of endotoxins, and the graphene concentration was determined. Concentration measurement was performed using the Beer–Lambert law, which relates absorbance to the product of the beam path length in meters (m), the concentration in grams per liter (g L^–1^), and the absorption coefficient. An absorption coefficient of 1390 L g^–1^ m^–1^ at 660 nm was used to determine the graphene concentrations.

### Atomic Force Microscopy (AFM)

Atomic force microscopy (AFM) was used to determine the lateral size and apparent thickness of the graphene flakes. A Dimension Icon (Billerica, MA) operating in peak-force mode was employed to conduct the AFM measurements. Samples were acquired from centrifuged graphene dispersions and were subsequently subjected to a 100-fold dilution before being drop-cast onto Si/SiO_2_ substrates.

The flake lateral size is found from the square root of the flake width multiplied by the flake length (eq 1). To estimate the total surface area in graphene samples, we calculated the surface area and volume of a corresponding disc (cylinder) with a radius equal to the half average lateral flake size and height equal to the measured peak height (eq 2).
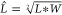
1where *L̂* is the lateral flake size, *L* is the flake length, and *W* is the flake length.
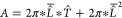
2where *A* is the approximated flake area,  is the average flake lateral size, and *T̂* is the peak flake thickness.

### Scanning Electron Microscopy (SEM)

A Magellan 400L (FEI Company, Hillsboro, OR) scanning electron microscope (SEM) was used to acquire images of the graphene samples to confirm the size measurements obtained through AFM. A field emission gun was run at a 6.3 pA current with an accelerating voltage of 5 kV. Images were captured in secondary electron detection mode.

### Raman Spectroscopy

Films of each graphene sample were drop cast onto silicon/silicon dioxide (Si/SiO_2_) substrate. Raman spectra were collected with an InVia micro-Raman spectrometer (Renishaw, Wotton-under-Edge, U.K.) at 514.5 nm and an incident power of below 1 mW to prevent potential damage.

### Cell Maintenance

The SW948 cells were cultured in Dulbecco’s modified Eagle medium with 5 mM glucose (DMEM), enriched with 2 mM l-glutamine, 10% fetal bovine serum (FBS), and antibiotics (penicillin at 100 U mL^–1^ and streptomycin at 100 μg mL^–1^). HUVECs were cultured in Endothelial Cell Growth Medium-2 (EGM-2) enriched using the EGM-2 Endothelial SingleQuots Kit according to the instructions provided by the manufacturer.

Both cell lines were maintained in a humidified incubator at 37 °C under a 5% CO_2_ atmosphere. Subculturing was conducted at approximately 80% confluency; for this, SW948 cells were detached using 0.5% trypsin–ethylenediaminetetraacetic acid (EDTA) solution, whereas a 0.05% trypsin–EDTA solution was used for the HUVECs. During this work, HUVECs were used until reaching eight passages, and then, new cells were defrosted.

### Cell Counting Kit-8 (CCK-8) Proliferation Assay

To test for cytotoxicity, 1.5 × 10^4^ cells of SW948 or HUVECs were seeded in 96-well plates in their respective culture media and incubated overnight. The various graphene suspensions were then added, and the cells were incubated for another 48 h. Following incubation, the cells were gently washed three times using phosphate-buffered saline (PBS), resuspended in a cell medium containing 10% of the cell proliferation reagent 8 (CCK-8, Dojindo Molecular Technologies, Kumamoto, Japan), and further incubated for 2 h. Metabolic activity was determined by measuring absorbance at 450 nm using a Spectramax Paradigm plate reader (Molecular Devices, Sunnyvale, CA), with a second absorbance measurement being performed at the same wavelength after washing the cells with PBS to determine background absorbance. The results were presented relative to controls of the respective cell lines.

### Zebrafish Larva Handling

Mature zebrafish were kept, and fertilized zebrafish eggs were obtained from the zebrafish facility at the University of Bergen, a facility run in accordance with the European Convention for the Protection of Vertebrate Animals Used for Experimental and Other Scientific Purposes. Following fertilization, zebrafish eggs, embryos, and larvae were incubated at 28.5 °C in an E3 medium (3 mM NaCl, 0.17 mM KCl, 0.33 mM MgSO_4_, and 10 μM methyl blue). During procedures like removal of the chorion, intravenous injections, and confocal imaging, zebrafish embryos and larvae were sedated using 0.7 mM of tricaine dissolved in the E3 medium. Prior to reaching 5 days post fertilization (dpf), the zebrafish larvae were euthanized by cooling on ice for 30 min followed by freezing overnight. Due to the euthanasia of zebrafish larvae prior to reaching 5 dpf, no approval from the national authority on research animals, the Norwegian Food Safety Authority, was necessary.

### Generation of a Zebrafish Line with Fluorescent Macrophages

A zebrafish line expressing fluorescent macrophages, Tg(mpeg1:mCherry), was created by injecting columned purified mpeg1:mCherry plasmid together with tol2 mRNA, at the one-cell stage of Casper zebrafish zygotes, following the protocol shown in ref (12). The Tol2-mpeg1-mcherry plasmid was a gift from Anna Huttenlocher (Addgene plasmid # 58935; http://n2t.net/addgene:58935; RRID:Addgene_58935). Larvae expressing fluorescent macrophages were selected, raised to adulthood, and crossed with wild-type casper to give the F1 generation. In this work, F2 zebrafish from in-crossed F1s were used for experiments.

### Toxicity and Biodistribution of Graphene in Zebrafish Larvae

To investigate the *in vivo* toxicity and biodistribution of graphene in zebrafish, graphene samples were first dispersed using bath sonication for 10 min. Casper zebrafish embryos at 2 dpf (in the long-pec stage^11^) were sedated, dechorionated, and placed on a 2% agarose bed. The embryos were then injected with approximately 4 nL of graphene solution into the posterior cardinal vein using a Narishige MMN-5 with MMO-220A (Narashige, Tokyo, Japan) micromanipulator system with an Eppendorf FemtoJet 4× microinjector (Eppendorf, Hamburg, Germany). Identical injections were performed for the solvents CMC and SDC to serve as controls together with noninjected larvae. The larvae were observed daily until euthanasia at 4 days postpartumption (dpf) to determine the mortality and any visual abnormalities, such as developmental defects and impacts on the cardiovascular system, following exposure. To visualize the agglomerations of graphene, larvae were imaged using bright-field microscopy, and the resulting images were stitched with the stitching plugin for FIJI (ver. 2.14.0) published by Preibisch et al.^14,15^

### Interactions between Intravenously Injected Graphene and Macrophages in Zebrafish Larvae

Tg(mpeg1:mCherry) zebrafish embryos with fluorescent macrophages were sedated and dechorionated at 2 dpf (in the long-pec stage^11^). To ensure consistency of the macrophage count and labeling in the larvae between experimental groups, zebrafish embryos were randomized into groups and imaged prior to sample injection. Confocal microscopy was performed using an Andor Dragonfly 505 confocal system (Andor Technology, Belfast, Northern Ireland) equipped with an inverted Nikon Ti-E microscope using a Nikon CFI Plan Apochromat lambda 10× objective (Nikon, Tokyo, Japan). The fluorescent macrophages were observed using a 561 nm excitation laser and a 575–625 nm bandpass filter. After initial imaging, the zebrafish embryos were injected with 4 nL of G-S-CMC (1 mg mL^–1^), G-M-CMC (6 mg mL^–1^), G-M-SDC (6 mg mL^–1^), or Milli-Q (MQ) water (Merck KGaA, Darmstadt, Germany) into the posterior cardinal vein as described for the toxicity and biodistribution studies. A separate control group was without injections. All larvae were imaged daily using confocal microscopy until the end of the experiment. Additional imaging using confocal microscopy was performed on the two following days. The resulting images were analyzed using the software tool presented in ref (16) to quantitate the macrophage populations. The resulting data was further processed using RStudio (version 2023.06.2) and FIJI (ver. 2.14.0).

### Complement System Activation

Activation of the complement system was measured as described in ref (17) using blood from healthy volunteers after informed consent and with approval of the regional committee for medical and health research ethics (REK SØR S-04114). In brief, graphene and stabilization agents were diluted in PBS with Ca and Mg to a concentration of 15 μg mL^–1^ for G-S-CMC, 120 and 600 μg mL^–1^ for the low and high concentrations, respectively, of both G-M-CMC and G-M-SDC, 300 μg mL^–1^ for CMC and 500 μg mL^–1^ for SDC. PBS with Ca and Mg served as the negative control. Blood was drawn from the volunteers, and lepirudin was added to a blood concentration of 50 μg mL^–1^, with lepirudin acting as a clotting inhibitor, but with no effect on the complement system.^18^ Immediately after obtaining the blood samples, 60 μL of the previously diluted samples and 300 μL of blood were combined and incubated for 30 min at 37 °C under mild shaking. The reaction was stopped using the addition of 7.2 μL of 0.51 M EDTA. After centrifugation at 3000 rcf for 20 min at 4 °C, plasma was separated and frozen at −80 °C until further analysis. The complement activation products C3bc (including C3b, iC3b, and C3c) and the terminal C5b-9 complement complex (TCC) were measured using highly specific mAbs to neoepitopes expressed specifically in the activation products and not in the native components. These in-house enzyme-linked immunosorbent assay have previously been described.^19^

## Results

### Graphene Characterization and Size Estimation

**Figure 1:**
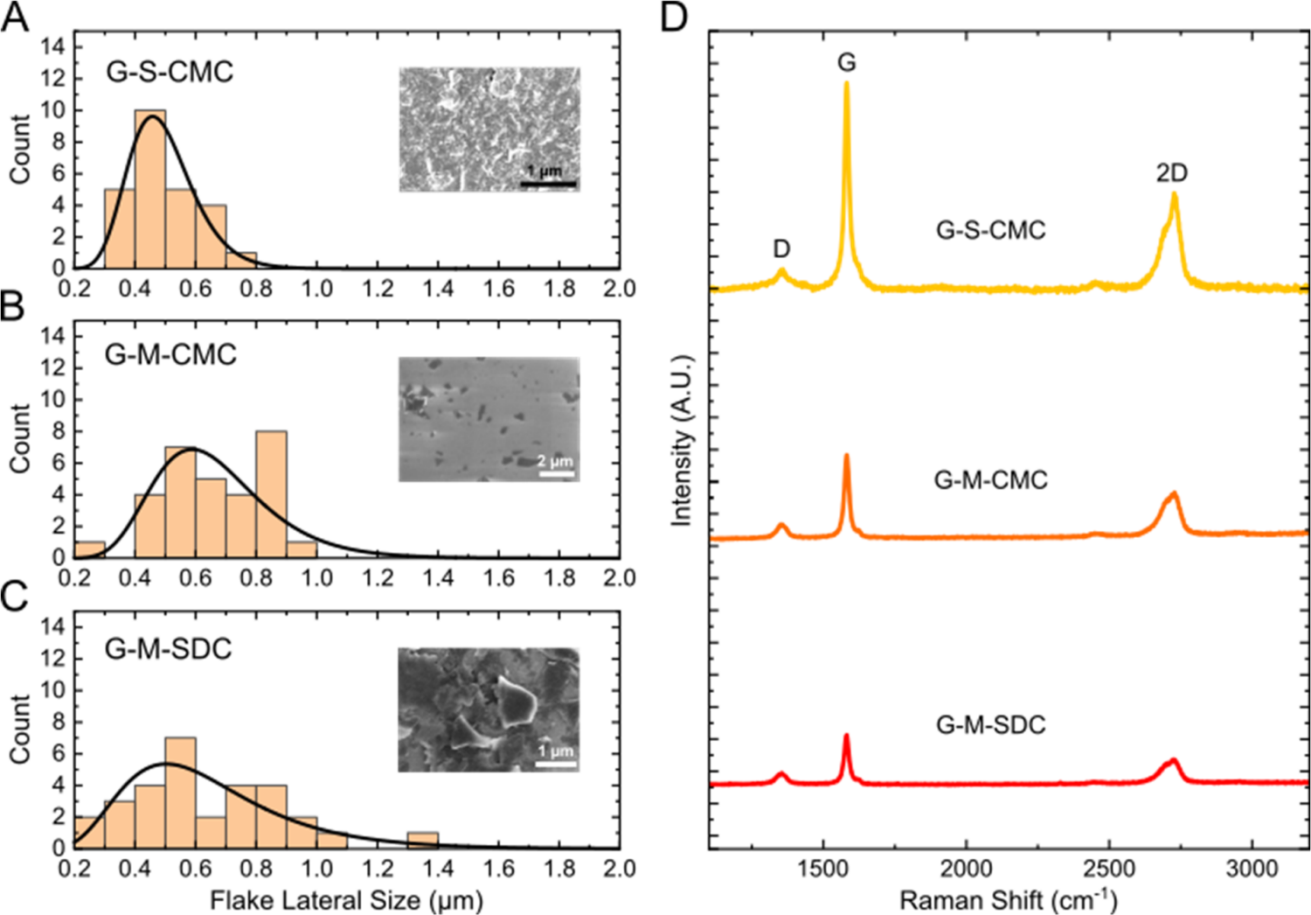
Characterization of graphene and size assessments. Three graphene samples using two different production methods, sonication (S) and microfluidization (M), with either solvent carboxymethylcellulose sodium salt (CMC) or sodium deoxycholate (SDC), were characterized using atomic force microscopy (AFM), scanning electron microscopy (SEM), and Raman spectroscopy. The lateral flake size measured using AFM is shown in (A)–(C), with cut-outs illustrating SEM imaging of the materials. Average lateral flake size and peak thickness (Figure S1). Raman spectroscopy was performed to confirm the production of graphene, as shown in (D).

Graphene samples were labeled G for graphene, S for sonication, or M for microfluidization and CMC for carboxymethylcellulose sodium salt or SDC for sodium deoxycholate. Size measurements were performed on all three materials using AFM (Figure 1A–C) and confirmed using SEM (examples of SEM imaging are shown in Figure S3). We found a comparable average lateral size of ∼0.5 μm and a peak thickness of 8–10 nm in all three samples (Table S1 for thickness measurements). For the microfluidized graphene samples, a small distribution of sheets with approximately 1 μm lateral size was also found. Using these measurements, the average sheet area, approximating the sheet geometry by a cylindrical disc with corresponding radius and height, was calculated as shown in Table S1, with a surface area per mg of material of 1.14 × 10^11^, 1.5 × 10^11^, and 9.16 × 10^10^ μm^2^ for G-S-CMC, G-M-CMC, and G-M-SDC, respectively.

Using Raman spectroscopy, we confirmed graphene production for all three samples. Figure 1D shows the spectra of each graphene sample and G, D, and 2D peaks, which are typical for few-layer graphene flakes. The Lorentzian-shaped 2D peak located at 2700 cm^–1^ indicates that the graphene comprised electronically decoupled layers.^20^

### *In Vitro* Cytotoxicity of the Graphene Samples in Cell Lines

**Figure 2:**
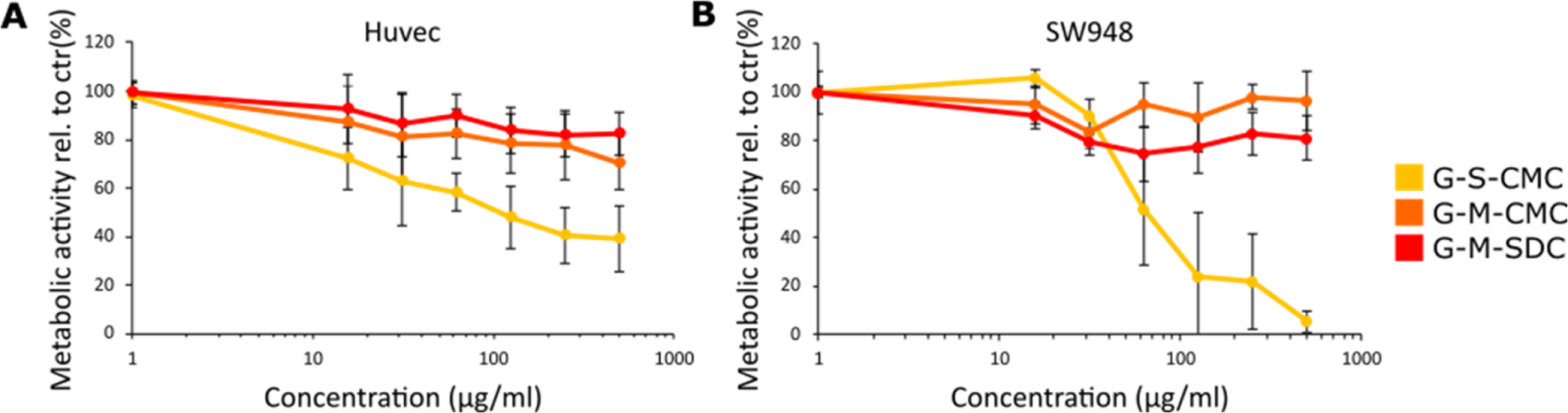
Graphene toxicity assessments using *in vitro* cell models. Cytotoxicity of the three graphene materials G-S-CMC, G-M-CMC, and G-M-SDC (with the abbreviations: G for graphene, S for sonication, CMC for carboxymethylcellulose sodium salt, and SDC for sodium deoxycholate) was evaluated using the CCK-8 metabolic based assay following 48 h of incubation. Here, metabolic activities in HUVECs (A) and SW948 (B) are plotted against increasing graphene concentrations to determine cell toxicity. Results are shown as mean ± standard deviation (SD); *N* = 9 and 5 for HUVECs and SW948, respectively.

The graphene samples were first tested for toxicity by measuring the metabolic activity using two human cell lines representing normal and malignant tissue types. After 48 h of incubation, the sonicated G-S-CMC was the most cytotoxic graphene material with an EC_50_ of 100 and 60 μg mL^–1^ for HUVECs (*N* = 9) and SW948 (*N* = 5), respectively (Figure 2). In comparison, G-M-CMC and G-M-SDC induced a modest reduction in the metabolic activity of approximately 20% at the highest concentration tested. It was further tested whether the toxicity was related to the stipulated surface area (Table S1) of the different graphene formulations. This did not change the EC_50_ value in any of the tested cell lines (data not shown).

### Activation of the Complement System by Graphene

**Figure 3:**
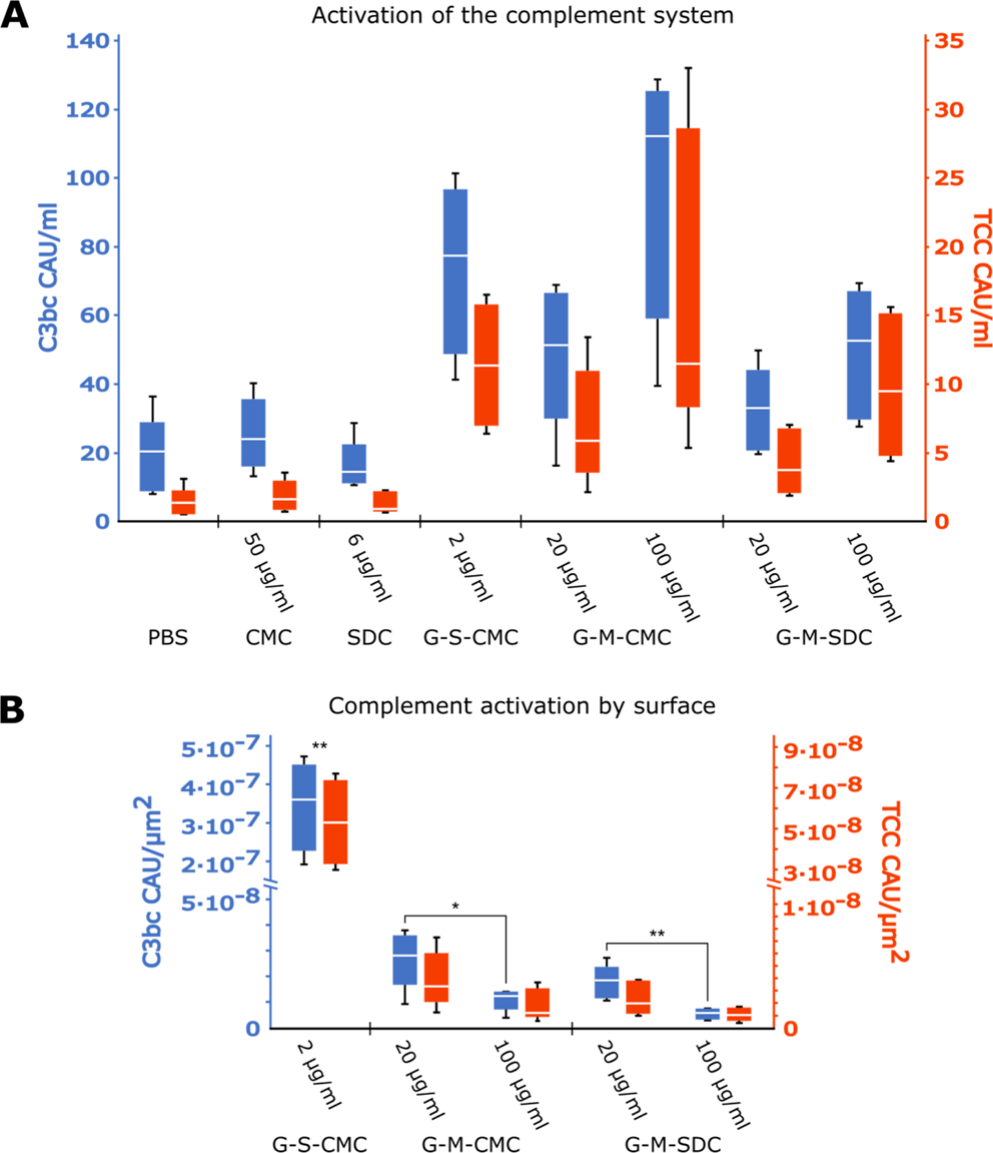
Complement activation by graphene samples. Concentrations of the activation products C3bc (blue) and TCC (red) are shown in complement arbitrary units (CAU) as previously used in ref (19). Complement activation relative to sample concentration is shown in (A) for the solvents (CMC) and (SDC), as well as the graphene samples, where sample names are constructed from G for graphene, the production method (S for sonication and M for microfluidization), followed by the solvent used (CMC or SDC). Activation relative to surface area is shown for the three graphene samples in (B). Here, the surface area was approximated based on cylindrical discs with a radius equivalent to half the measured average lateral size and height equivalent to the peak thickness in each graphene sample (Table S1). Significance in (B) was calculated using a two-tailed *t* test with *p < 0.05 and **p < 0.01, with results for G-S-CMC in (B) being significant with at least ** to the respective measurements of all other samples.

Complement system activation was determined by measuring the markers C3bc and TCC in whole blood samples after 30 min incubation with the samples or a negative PBS control. Activation relative to sample concentrations is shown in Figure 3A. Compared to the PBS control, 50 μg mL^–1^ CMC and 6 μg mL^–1^ SDC did not lead to a significant activation of the complement system. G-S-CMC at 2 μg mL^–1^ induced a marked complement activation as detected by both C3bc and TCC, comparable to G-M-CMC and G-M-SDC when reaching a 10-fold higher concentration (20 μg mL^–1^); the concentration of the latter two (microfluidization) was increased to 100 μg mL^–1^, still in the range of the same level as G-S-CMC at 2 μg mL^–1^. Activation of the complement system was also calculated as a function of the estimated surfaces of the nanoflakes (Figure 3B). Here, G-S-CMC led to a significantly higher level of both C3bc and TCC per μm^2^ surface when compared to G-M-CMC and G-M-SDC. For the microfluidized graphene samples, we found an inverse correlation between sample concentration and C3bc but not TCC.

### Graphene Toxicity in the Zebrafish Larvae

To establish the toxicity of the graphene samples *in vivo*, we exposed zebrafish larvae to graphene suspensions and solvents through one 4 nL intravenous injection into the posterior cardinal vein at the two days post fertilization (dpf) stage. For each of the samples, as well as a MQ water control, 10 larvae were injected. F2or MQ water, the two microfluidized graphene samples, and the solvent CMC, injections of 6 mg mL^–1^ for graphene and 3 mg mL^–1^ for CMC did not lead to death prior to reaching euthanasia at 5 dpf. Injections of G-S-CMC (148 μg mL^–1^) and SDC (5 mg mL^–1^) led to two and one death at 1 day post injection, respectively.

**Figure 4:**
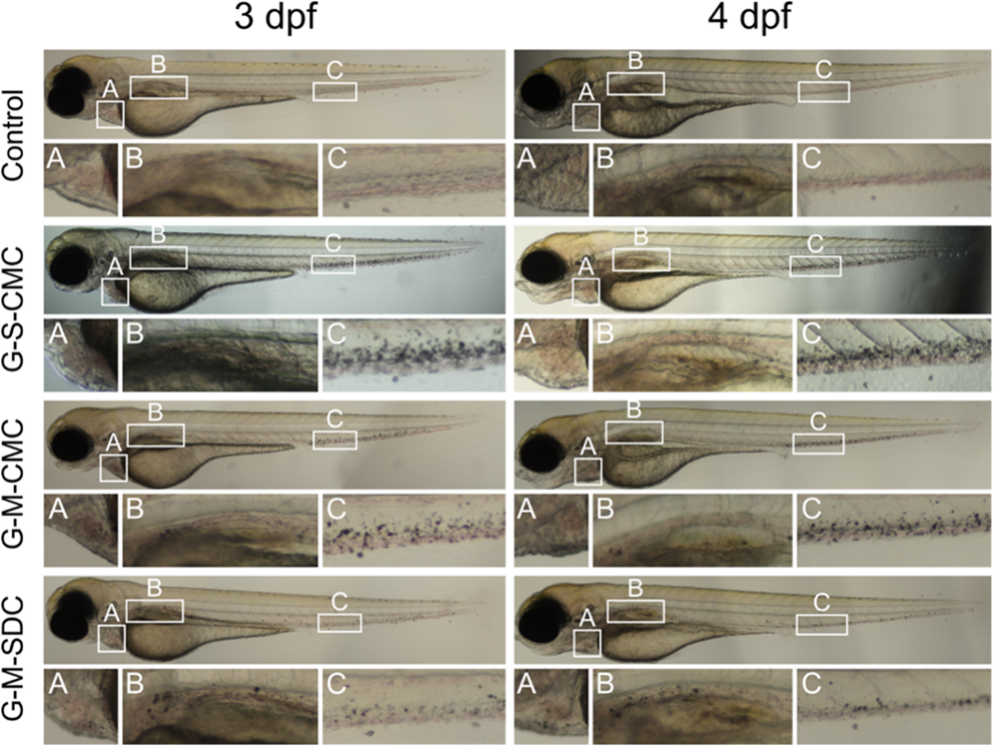
Agglomeration of graphene in zebrafish larvae. Zebrafish larvae were injected intravenously 2 days post fertilization (dpf) with 4 nL of 1 mg mL^–1^ G-S-CMC or 6 mg mL^–1^ of either G-M-CMC or G-M-SDC injections of graphene solution or left without injection to serve as control. Four larvae for each group were imaged 3 and 4 days post injection. Representative images of the selected single larva in each of the treatment groups; control, G-S-CMC, G-M-CMC, and G-M-SDC, are shown. Agglomerations were found predominantly in the ventral tail area (C), with some additional agglomeration in the heart (A) and area downstream of injection site (B), as shown below each larva.

An additional experiment was performed to characterize visual toxic effects, such as morphological and developmental abnormalities. Here, four zebrafish larvae per graphene sample were exposed to 4 nL of intravenous injection at 2 dpf and imaging on days 3 and 4. Concentrations of the graphene samples were 1 mg mL^–1^ for G-S-CMC and 6 mg mL^–1^ for G-M-CMC and G-M-SDC. Injected larvae were compared to larvae without injections as a control. No morphological abnormalities were observed in any of the injected larvae. Graphene agglomerates were found to accumulate in mainly three regions for all three samples: downstream of the injection site, the heart, and the ventral region of the tail (Figure 4).

### Macrophage Response to Graphene in Zebrafish Larvae

**Figure 5:**
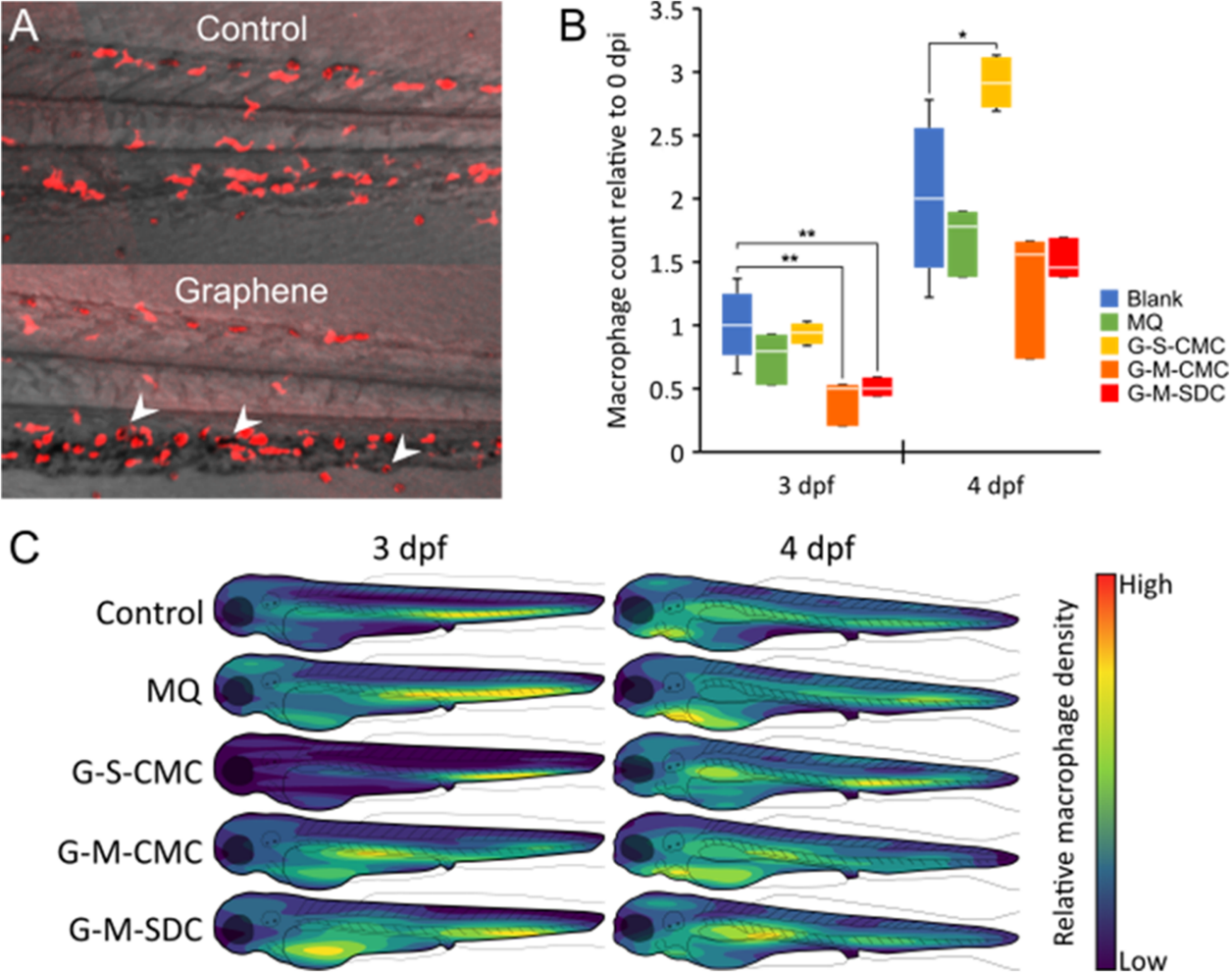
Macrophage count and position in graphene injected zebrafish larvae. Zebrafish larvae with fluorescent macrophages were intravenously injected at 2 days post fertilization (dpf) with Milli-Q (MQ) water, G-S-CMC (1 mg mL^–1^, *N* = 4), G-M-CMC (6 mg mL^–1^, *N* = 3), and G-M-SDC (6 mg mL^–1^, *N* = 3) or left without injection (*N* = 6). The larvae were imaged daily using confocal microscopy from the day of injection, until 4 dpf. Image (A) shows a cut-out of the ventral tail region from a larva injected with G-M-CMC the day following injection (3 dpf). Macrophages can be seen in red, with white arrows indicating macrophages engulfing graphene agglomerations. For illustration purposes, the red fluorescent channel was flattened using a max-projection while the bright-field channel was flattened using stack-focusing. Using a software tool,^16^ the count of macrophages was determined from the day of injection (2 dpf) to 2 days post injection (4 dpf). The change in macrophage count relative to the day of injection is shown for 3 and 4 dpf (B). Using the location of each cell detected in the confocal images, cell density heatmaps combining all larvae in each group were created (C). Significance in B was calculated using a two-tailed *t* test with *p < 0.05 and **p < 0.01.

To assess any macrophage response to graphene flakes, transgenic larvae expressing fluorescent macrophages were intravenously injected with 4 nL of either graphene suspension or MQ at 2 dpf (*N* = 3 for all groups except for G-S-CMC with *N* = 4), left without injection (*N* = 6), and imaged daily using confocal microscopy until reaching 4 dpf. The concentrations were equal to that of the biodistribution experiment at 1 mg mL^–1^ for G-S-CMC and 6 mg mL^–1^ for G-M-CMC and G-M-SDC. Signs of phagocytosis were observed in all three graphene samples (Figure 5A). Using our software tool described in ref (16), macrophage populations were segmented from the confocal images and tracked over the duration of the experiment. The number of detected macrophages relative to the count prior to injection is shown in Figure 5B. A significant reduction in macrophage count was observed for microfluidized graphene samples 1 day after injection, whereas the following day, the macrophage counts returned to equal that of the control samples. For G-S-CMC, the macrophage count was equal to the control samples at 3 dpf; however, the next day a significant increase relative to the noninjected control was observed.

The distributions of macrophages were also mapped for each day of the experiment (Figure 5C). Here, each heat map displays the relative macrophage density within each group from the highest (yellow) to the lowest (dark blue). Throughout the experiment, both controls exhibit comparable distributions. Larvae exposed to G-S-CMC showed elevated macrophage density, mainly in the ventral tail area, 1 day after injection. However, the following day, the macrophage distribution returned to a distribution comparable to controls, albeit with a slight elevation in the ventral tail area and lower count around the heart. Larvae exposed to G-M-CMC exhibited a slightly altered distribution compared to controls at 3 dpf. A comparatively high concentration was found around the site of injection, with a slightly lower concentration in the ventral tail area. At 4 dpf, the macrophage distributions returned to a similar distribution, as seen in the controls. Compared to the control groups, larvae exposed to G-M-SDC, macrophages were found to accumulate in larger concentrations around the heart/yolk sac, in addition to the ventral tail area. At 4 dpf, the highest concentration of macrophages was found around the injection site in the posterior cardinal vein.

## Discussion

The present study provides novel insights into the two production methods, sonication and microfluidization, of graphene flakes of similar dimensions with respect to their structure and their effect on biocompatibility as evaluated by established techniques for cellular toxicity, macrophage response, and complement activation.

Both production methods yielded graphene with sheets of similar dimensions. While the size distributions of G-M-SDC indicated a wider dispersity compared to sonicated graphene, with some sheet sizes around 1 μm, average lateral sizes were comparable. Our simplified approach to estimating the total surface area assumed the graphene sheets to be disc-shaped in order to compensate for the slight deviations in the lateral size between the graphene samples and facilitate the interpretation of the subsequent experiments. Initial toxicity experiments on human cell lines showed that the G-S-CMC decreased viability in cells at concentrations of 100 and 60 μg mL^–1^ for HUVECs and SW948, respectively, whereas this decrease in viability was not found for the two other graphene samples. A possible explanation for this discrepancy in cytotoxicity could be the previously reported defects generated during the sonication process, which increases the number of oxygen-rich sites in the material and consequently exposes cells to higher oxidative stress.^7,8^ While this has mostly been studied using graphene oxide, with comparatively more defects in the carbon lattice than our graphene samples, we suspect that the graphene sonication process induces more defect sites and thus may impose higher oxidative stress exposure for the cells treated with the G-S-CMC.

G-S-CMC also led to significantly higher activation of the complement system when compared to that of microfluidized graphene. This indicates that the two processes generate a surface that from the complement potency differs substantially. The complement system distinguishes self from nonself in a unique manner.^21^ If a surface has properties similar to that of the host, the alternative complement pathway will be only slightly activated. In contrast, if a surface is not recognized as self, an immediate activation of the alternative pathway will occur since factor B will be preferred to bind to C3b instead of factor H and the amplification loop will start. This is the most likely explanation for the major differences seen between sonication and microfluidization with respect to complement activation in the present study.

In both microfluidized graphene samples, the lower concentration of 20 μg mL^–1^ surprisingly showed a slightly higher complement activation relative to surface area compared to samples with 100 μg mL^–1^. An explanation for this could be an increased agglomeration of graphene in the more concentrated samples. Agglomeration will reduce the available surface area compared to the total surface area of graphene, and the complement system is thus presented with less surface area for reaction. In cell toxicity assays, however, cytotoxicity relative to the available graphene surface did not increase at lower concentrations. Thus, different mechanisms may underlie cytotoxicity and complement system activation.

No apparent toxic effects were observed in zebrafish larvae after intravenous injections of graphene from either production method. Even though large graphene agglomerates were observed, the larvae developed normally and only three of a total of 60 larvae died, two after G-S-CMC and one after the SDC stabilization agent injections. The low number of deaths could be due to factors not related to graphene exposure, for example, mechanical damage after inadvertent poor handling. Due to the high tolerance to the graphene samples, we were unable to determine the LD_50_ or maximum tolerable dose. The graphene biodistributions were also found to be similar across all three samples. While the graphene agglomerations around the larval heart did not lead to any observed toxicity in our study, previous research has found a cardiotoxic effect of related graphene materials in mice.^22^ These findings indicate that prolonged exposure could lead to adverse effects in the zebrafish larva model as well and warrant further investigation.

Utilizing zebrafish with fluorescent macrophages enabled the visualization of graphene phagocytosis. Despite these observations, no significant change in the total amount of graphene inside the larvae was found. While this could be due to the relatively short duration of our experiments, the zebrafish larva model with fluorescent macrophages could be a valuable model to investigate graphene clearance in future studies. The graphene production methods impacted macrophage count and distribution in larvae differently. For the microfluidized graphene samples, we observed a significant reduction in macrophage counts the day after injection compared to the noninjected control; however, this returned to comparable levels to the control the following day. A reason for this could be acute macrophage death caused by graphene, but another likely explanation is through fluorescent quenching and absorption by graphene, thus artificially lowering the count of detected macrophages, as their diminished fluorescent signal was undistinguishable from background fluorescence. This reduction was not found to be significant when compared to the control sample injected with MQ water, which itself showed a slight, nonsignificant reduction compared to control larvae without injection. Hence, the injection process itself could have contributed to lowering the macrophage count, for instance, through increased stress. Interestingly, exposure to G-S-CMC had the opposite effect, where the macrophage counts significantly increased compared to both control groups 2 days after injection. This is in line with our findings that the graphene production method affects immune response by complement activation.

The biodistributions of macrophages in larvae exposed to G-S-CMC were different compared with either control group. While the largest concentration of macrophages was found in the ventral tail area of the larvae injected with sonicated microfluidized graphene, in larvae injected with G-S-CMC we found that surrounding areas of the larva exhibited comparatively lower macrophage concentrations. Importantly, the ventral tail area was also where most of the G-S-CMC agglomerates were found (Figure 4). Graphene could induce the recruitment of macrophages through local cytotoxic effects, similar to the macrophage recruitment after cellular or tissue injury of zebrafish larvae, and/or through activation of the complement system, which was shown to be activated following the injection of polystyrene nanoparticles in zebrafish embryos.^23,24^ The higher cytotoxicity and complement activation of G-S-CMC could hence explain the large shift in the macrophage distribution. For microfluidized graphene, changes in the macrophage distribution were less pronounced with agglomerates around the injection site and the heart, in combination with injuries following injection, potentially leading to an increased presence of macrophages in these areas.

## Conclusions

Upscaling graphene production methods to leverage their use beyond small laboratory experiments is important. The choice of production method will impact the quality of the graphene, which results in different graphene characteristics that are important to evaluate if this material is to be used for biomedical use. Neither sonicated nor microfluidized graphene materials showed acute toxic effects in zebrafish larvae. However, the sonicated G-S-CMC showed increased cytotoxicity and complement activation and had a larger impact on macrophage distribution when compared to both microfluidized materials. With microfluidization being a commercially scalable approach to graphene production, these findings further support the use of this method to produce graphene for biomedical applications with minimal impact on biological processes.

## Supporting Information Available

Height measurements of graphene samples (Figure S1); cytotoxic effects of graphene relative to surface area (Figure S2); scanning electron images of the three graphene materials (Figure S3); G-M-CMC, G-M-SDC, and G-S-CMC (Table S1) (PDF)
