# Supplementary material for: Impact of the Graphene Production Methods Sonication and Microfluidization on In Vitro and In Vivo Toxicity, Macrophage Response, and Complement Activation
Source: ACS Omega. 2024 Sep 18;9(39):40468–76. doi: 10.1021/acsomega.4c03189 (PMC11447947; doi:10.1021/acsomega.4c03189)
Supplement: Supplementary file 1 — ao4c03189_si_001.pdf [file ao4c03189_si_001.pdf]

Impact of the graphene production methods sonication and microfluidization on *in vitro* and *in vivo* toxicity, macrophage response and complement activation

Jan-Lukas Førde[1,2], Abdelnour Alhourani[3], Tian Carey[4§], Adrees Arbab[4], Kari Espolin Fladmark[5], Silje Skrede[6,7], Tom Eirik Mollnes[8,9], Lars Herfindal[2], Hanne Røland Hagland[3*]

[1] Department of Internal Medicine, Haukeland University Hospital, Bergen, Norway
[2] Centre for Pharmacy, Department of Clinical Science, University of Bergen, Bergen, Norway
[3] Department of Chemistry, Bioscience and Environmental Engineering, University of Stavanger, Stavanger, Norway.
[4] Textile Two Dimensional Ltd
[5] Department of Biological Sciences, University of Bergen, Bergen, Norway.
[6] Section of Clinical Pharmacology, Department of Medical Biochemistry and Pharmacology, Haukeland University Hospital, Bergen, Norway
[7] Department of Clinical Science, University of Bergen, Bergen, Norway
[8] Research Laboratory, Nordland Hospital Trust, Bodø, Norway
[9] Department of Immunology, Oslo University Hospital and University of Oslo, Oslo, Norway.
[§] Present address: School of Physics, CRANN & AMBER Research Centre, Trinity College, Dublin 2, Ireland

*Corresponding author: hanne.r.hagland@uis.no

Supporting information

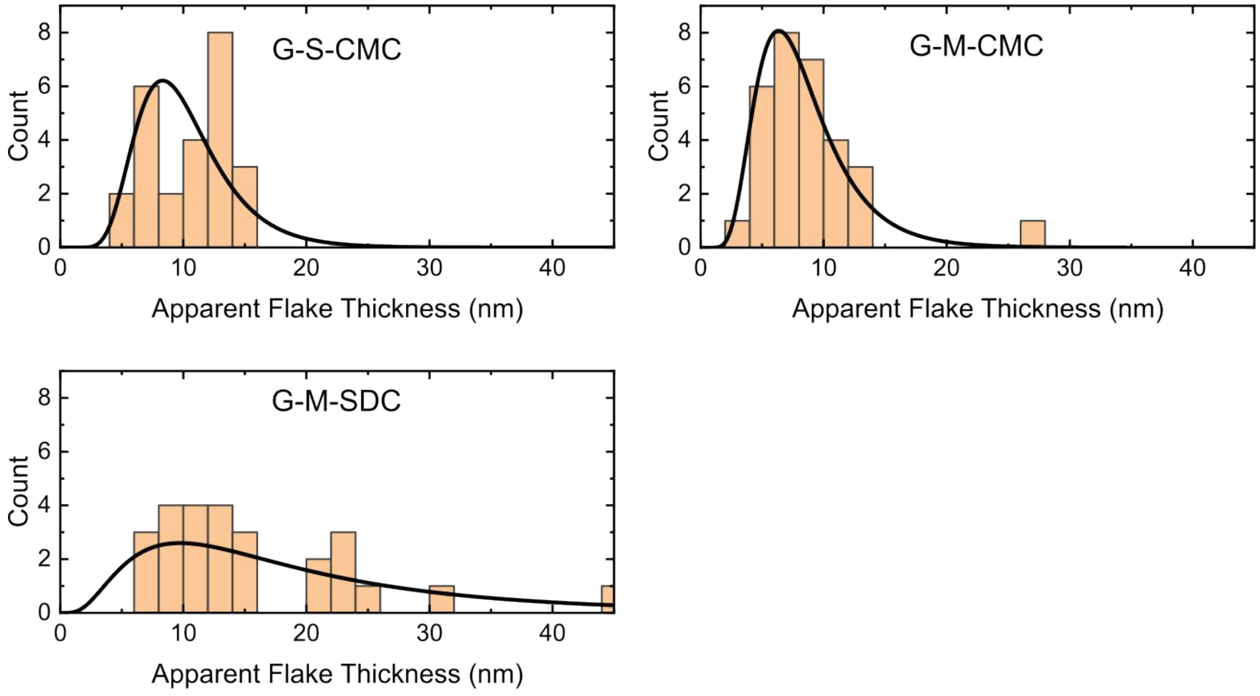

Figure S1 – Height measurement of graphene samples. Three graphene samples using the two different production methods sonication (S), and microfluidization (M), and the two different stabilization agents carboxymethylcellulose sodium salt (CMC) and sodium deoxycholate (SDC) were

characterized using atomic force microscopy (AFM). The plots illustrate the measured apparent thicknesses of the graphene sheets.

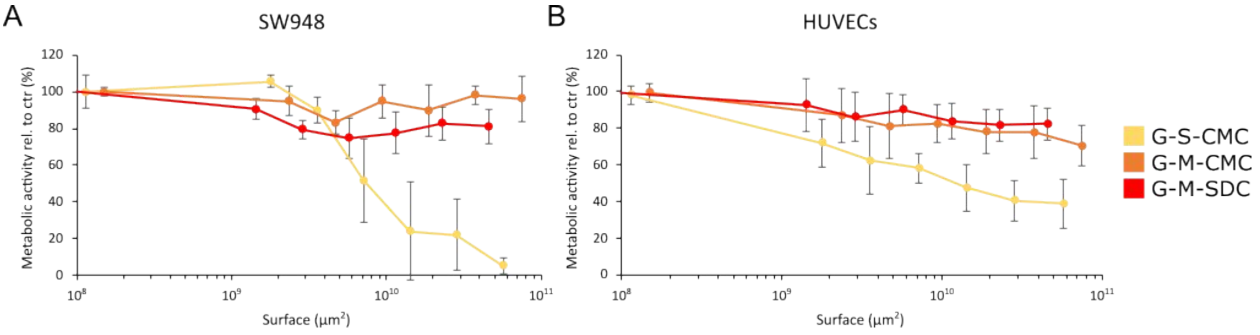

Figure S2 – Cytotoxic effects of graphene relative to surface area. Cytotoxicity of the three graphene materials G-S-CMC, G-M-CMC, and G-M-SDC (with the abbreviations: G for graphene, S for sonication, CMC for carboxymethylcellulose sodium salt, and SDC for sodium deoxycholate) was evaluated using a CCK-8 assay following 48 h incubation. Here, metabolic activity in HUVECs (A) and SW948 (B) are plotted against the calculated graphene surface area (Figure 1D). Results are shown as mean ± SD; N = 9 and 5 for HUVECs and SW948, respectively.

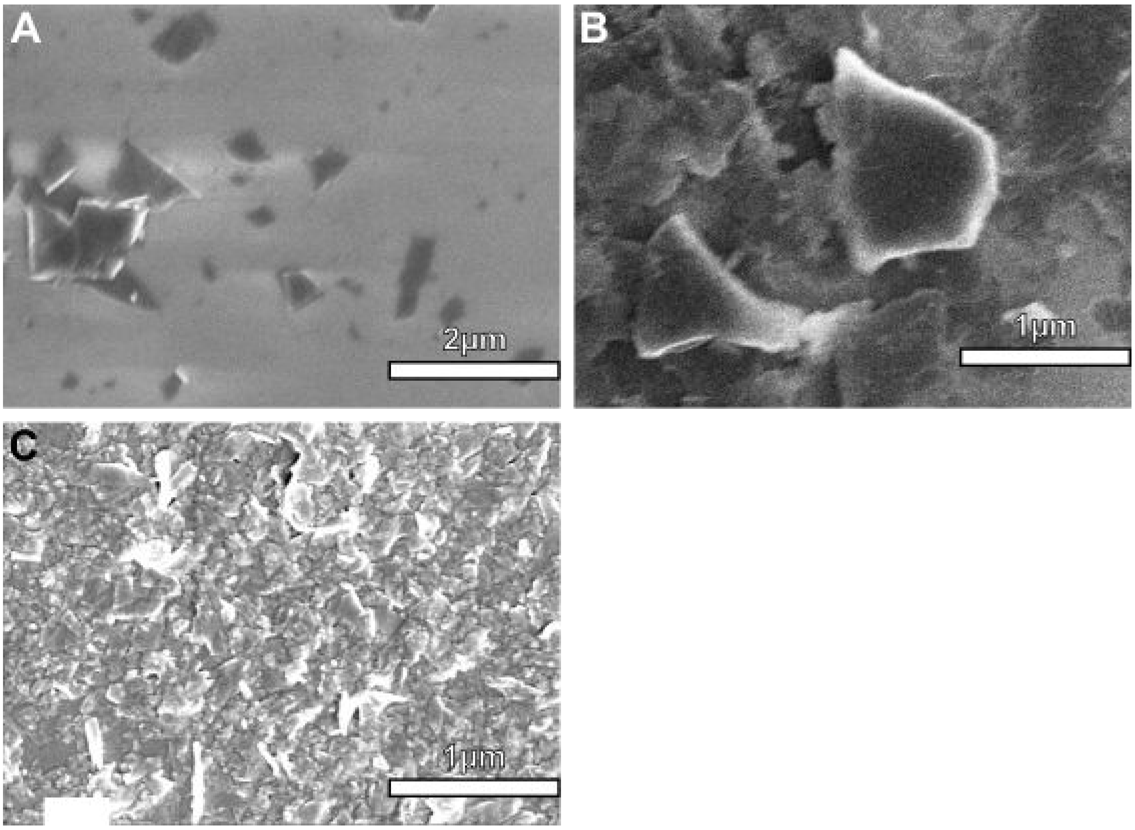

Figure S3 – SEM imaging of graphene materials. The three materials G-M-CMC (A), G-M-SDC (B) and G-S-CMC (C) were imaged using a Magellan 400L scanning electron microscope (SEM) to confirm the size measurements obtained through AFM. The field emission gun was run at 6.3 pA current with an accelerating voltage of 5 kV. Images were captured in secondary electron detection mode.

Sample	Avg. lat. size (µm)	Peak thickness (µm)	Cylinder area (µm^2) $2\pi hr+2\mu r^2$	
			Per sheet	Per mg
**G-S-CMC**	**0.49**	**8**	**0.39**	**$1.14\cdot10^{11}$**
**G-M-CMC**	**0.55**	**6**	**0.49**	**$1.50\cdot10^{11}$**
**G-M-SDC**	**0.52**	**10**	**0.44**	**$9.16\cdot10^{10}$**

Table S1 – Approximation of graphene surface area from apparent flake thickness and average lateral size. Three graphene samples using the two different production methods sonication (S), and microfluidization (M), and the two different solvents carboxymethylcellulose sodium salt (CMC) and sodium deoxycholate (SDC) were characterized using atomic force microscopy (AFM). Using the measured peak flake thickness (Figure S1) and average lateral size (Figure 1A-C)
